# Tumor vessel normalization after aerobic exercise enhances chemotherapeutic efficacy

**DOI:** 10.18632/oncotarget.11748

**Published:** 2016-08-31

**Authors:** Keri L. Schadler, Nicholas J. Thomas, Peter A. Galie, Dong Ha Bhang, Kerry C. Roby, Prince Addai, Jacob E. Till, Kathleen Sturgeon, Alexander Zaslavsky, Christopher S. Chen, Sandra Ryeom

**Affiliations:** ^1^ Department of Cancer Biology, Perelman School of Medicine, University of Pennsylvania, Abramson Family Cancer Research Institute, Philadelphia, PA 19104, USA; ^2^ Department of Physiology, University of Pennsylvania, Philadelphia, PA 19104, USA; ^3^ Department of Bioengineering, Boston University, Boston, MA 02215, USA

**Keywords:** tumor vascular normalization, NFAT, thrombospondin-1, exercise

## Abstract

Targeted therapies aimed at tumor vasculature are utilized in combination with chemotherapy to improve drug delivery and efficacy after tumor vascular normalization. Tumor vessels are highly disorganized with disrupted blood flow impeding drug delivery to cancer cells. Although pharmacologic anti-angiogenic therapy can remodel and normalize tumor vessels, there is a limited window of efficacy and these drugs are associated with severe side effects necessitating alternatives for vascular normalization. Recently, moderate aerobic exercise has been shown to induce vascular normalization in mouse models. Here, we provide a mechanistic explanation for the tumor vascular normalization induced by exercise. Shear stress, the mechanical stimuli exerted on endothelial cells by blood flow, modulates vascular integrity. Increasing vascular shear stress through aerobic exercise can alter and remodel blood vessels in normal tissues. Our data in mouse models indicate that activation of calcineurin-NFAT-TSP1 signaling in endothelial cells plays a critical role in exercise-induced shear stress mediated tumor vessel remodeling. We show that moderate aerobic exercise with chemotherapy caused a significantly greater decrease in tumor growth than chemotherapy alone through improved chemotherapy delivery after tumor vascular normalization. Our work suggests that the vascular normalizing effects of aerobic exercise can be an effective chemotherapy adjuvant.

## INTRODUCTION

Tumor vasculature is unorganized and leaky; as many as 50% of tumor vessels are non-functional [1]. Consequently, drug delivery to tumors is inefficient, reducing chemotherapeutic efficacy. The concept of vascular normalization, originally proposed by Jain and colleagues in 2001, posits that restoring the balance of angiogenic regulators by decreasing pro-angiogenic factors remodels tumor vasculature to become more organized and functional, similar to normal vasculature [2]. Anti-angiogenic drugs normalize tumor vasculature to increase chemotherapy delivery to the tumor, and have been used in combination with chemotherapy to enhance chemotherapeutic efficacy in multiple cancer types [3]. For example, the utility of vascular normalization has been demonstrated in glioblastoma patients who were treated with the anti-angiogenic agent cediranib in combination with chemoradiation. Improved tumor blood perfusion after cediranib treatment correlated with improved overall survival [4]. However, resistance to anti-angiogenic therapy and significant adverse side effects necessitate better approaches to normalize tumor vasculature [5].

Since all drugs have accompanying side effects, non-pharmacologic approaches for tumor vascular normalization would be ideal. In addition to growth factor signaling, blood vessel growth is regulated by mechanical cues created by changes in blood flow, velocity and pressure. Blood flow induces shear stress, the mechanical force on endothelium that is critical for the establishment of mature, functional vasculature during development. Thus, we reasoned that increasing blood flow in tumor vasculature may promote tumor vessel function. One approach to modulate blood flow systemically is through aerobic exercise. Aerobic exercise has been shown to be safe in patients undergoing chemotherapy and to improve quality of life measures such as mobility and fatigue [6, 7]. Recently, tumor vascular remodeling in response to exercise has been described in mouse cancer models, but the molecular mechanisms regulating the tumor vascular response to exercise have not been elucidated [8–10]. In this work, we utilized moderate intensity treadmill running to increased blood flow in mice with B16F10 melanoma or PDAC-4662 pancreatic ductal adenocarcinoma tumors. Tumor growth was significantly inhibited in mice treated with chemotherapy and subjected to aerobic exercise as compared to mice treated with chemotherapy alone. Aerobic exercise triggered tumor vascular normalization, characterized by increased vascular length and perfusion and increased chemotherapy delivery to the tumor bed. Utilizing microfluidic devices and modified tissue culture dishes to mimic increased blood flow-induced shear stress *in vitro*, we found that shear stress alters the angiogenic secretome of endothelial cells (ECs), leading to secretion of soluble factors that inhibit tumor EC sprouting and permit vascular remodeling. Further, we demonstrate that fluid flow activates the transcription factor Nuclear Factor of Activated T cells (NFAT) in ECs, modulating the expression of multiple pro- and anti-angiogenic factors [11, 12]. Our studies identified increased levels of thrombospondin-1 (TSP-1), a key anti-angiogenic protein, as a critical downstream mediator of tumor vascular normalization during exercise via calcineurin-NFAT-TSP-1 signaling.

## RESULTS

### Exercise normalizes tumor vasculature

**Figure 1:**
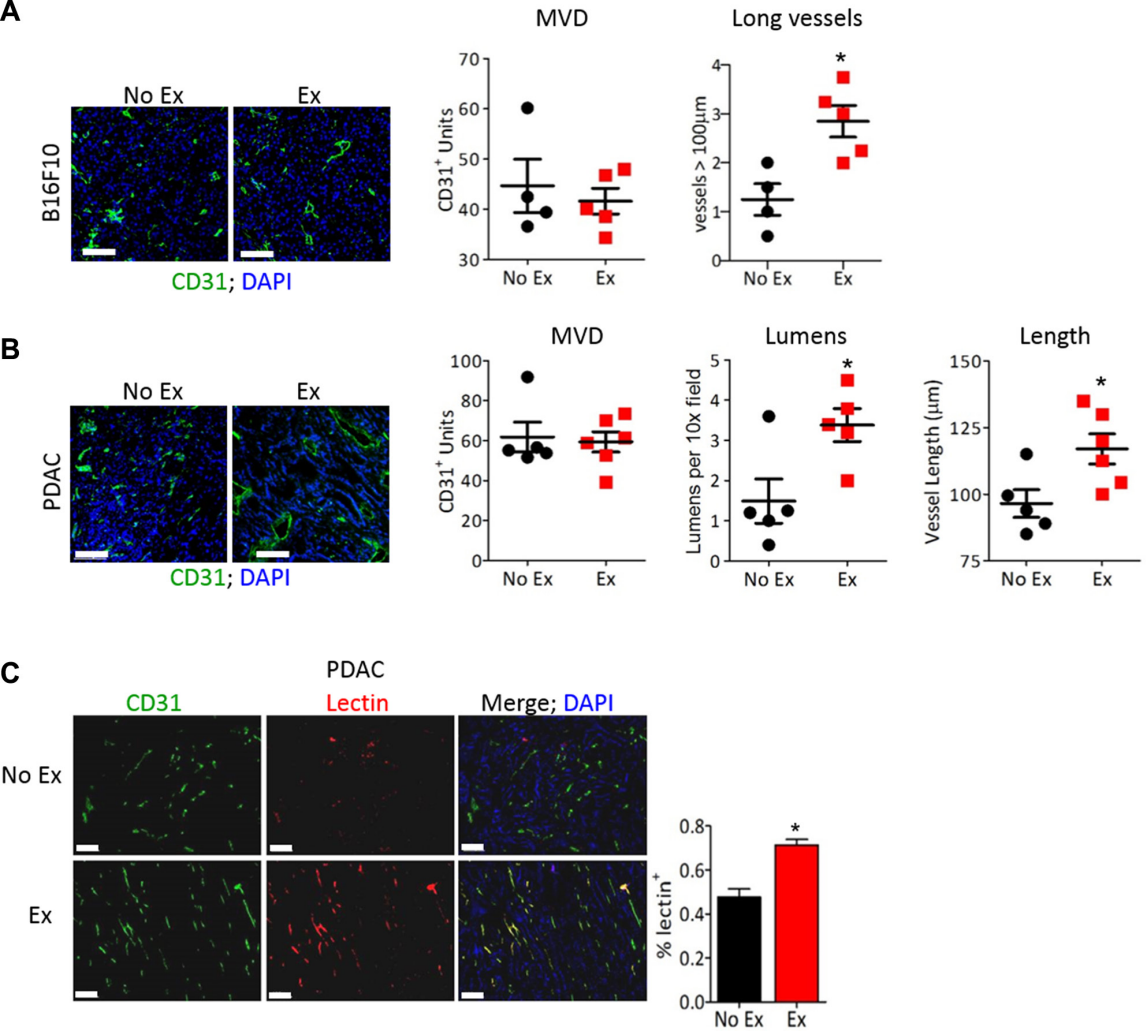
Exercise normalizes tumor vasculature (**A**, **B**) Representative images of anti-CD31 immunofluorescence on (A) B16F10 or (B) PDAC-4662 tumors with DAPI staining to visualize the nucleus. After B16F10 or PDAC-4662 tumors were palpable, mice were randomized into treadmill running or no exercise groups. 21 days later, tumors were harvested. Microvessel density (MVD), the number of vessels > 100 μm (large), the number of visible lumens and the average vessel length, and were counted in 5 random sections/tumor and averaged to obtain a value per tumor, *p < 0.05, *n* = 5–6 per group, Bars = 100 μm. (**C**) Representative images of anti-CD31 immunofluorescence and isolectin-B4 positive vessels from PDAC-4662 tumors. When tumors were palpable, PDAC-4662 tumor-bearing mice were treated with gemcitabine with or without daily treadmill running for two weeks. Prior to euthanasia, mice were injected with isolectin-B4. Quantification of Isolectin-B4 (red) and CD31 (green) double positive vessels is shown on the right with double positive vessels quantified in 5 sections/tumor and shown as the mean +/− S.E.M., *n* = 4 per group, *p < 0.05, Bars = 100 μm.

One of the many effects of aerobic exercise is a systemic increase in blood flow that increases the mechanical force on endothelial cells at a rate proportional to exercise intensity [13]. Mechanical forces have been shown to promote mature, functional vasculature in healthy tissues. We therefore used moderate intensity (60–70% VO_2_ max [14]) daily treadmill running in tumor-bearing mice to examine the effect of exercise-increased blood flow on tumor vasculature. Tumor vascular normalization includes a decrease in non-functional sprouts, an increase in hierarchical vasculature with reduced leakiness, and no change in microvessel density, resulting in increased blood delivery [2]. Consistent with this, B16F10 tumors from exercised mice showed no change in microvessel density but a significant increase in longer vessels. Exercise shifted the morphology of tumor vasculature from disconnected, small clumps of CD31^+^ endothelial cells (ECs) to elongated blood vessels (Figure 1A). Similarly, the average vessel length and number of visible lumens was significantly higher in PDAC-4662 tumors from exercised compared to non-exercised mice (Figure 1B). The hallmark of vascular normalization is improved vessel function that can be quantified by lectin perfusion. We identified a 24% increase in lectin-positive (functional) vessels in PDAC-4662 tumors from exercised compared to non-exercised mice (Figure 1C).

### Exercise enhances chemotherapeutic efficacy

**Figure 2:**
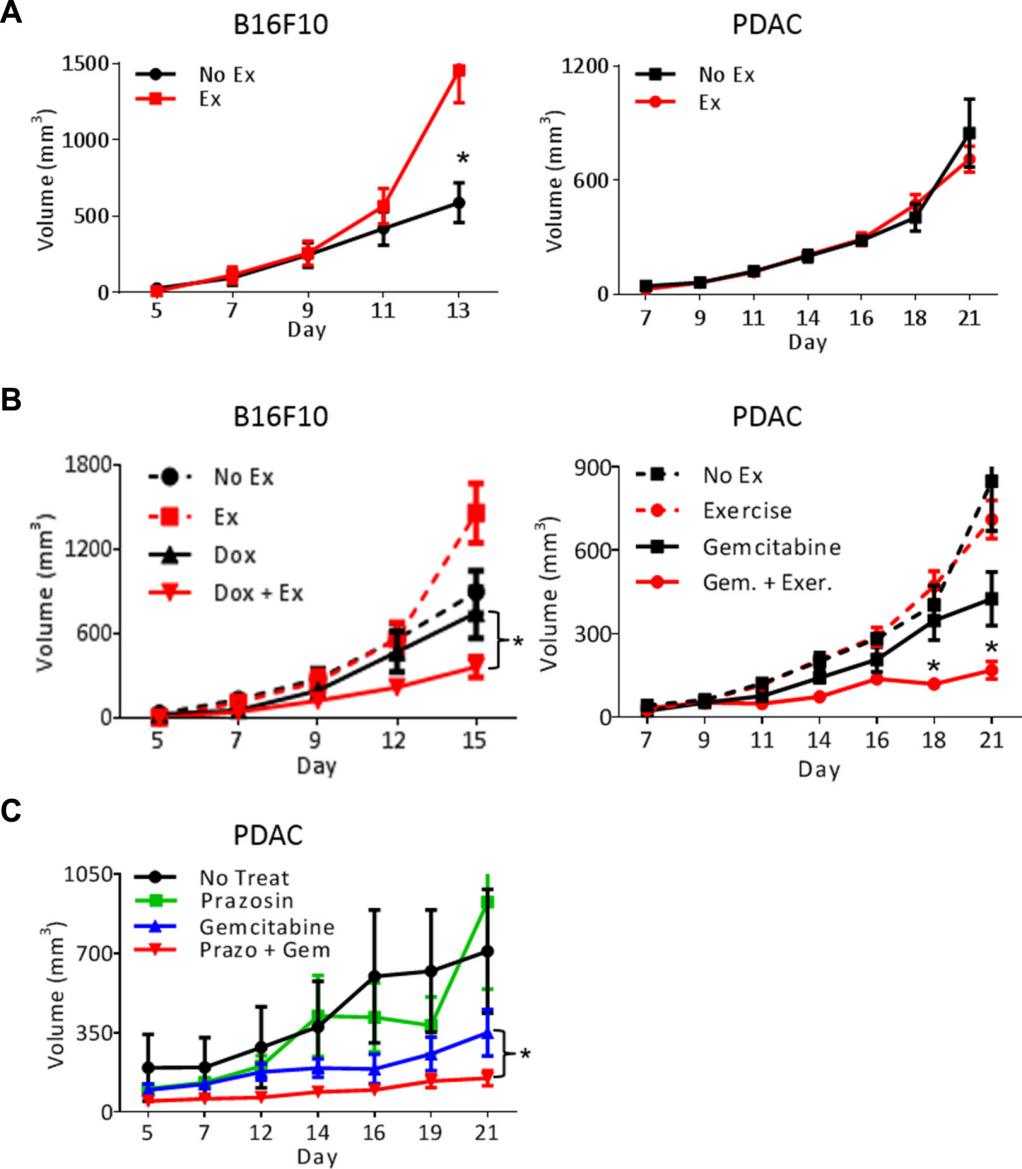
Exercise-induced shear stress increases chemotherapeutic efficacy (**A**) Tumor growth in exercised or non-exercised mice. B16F10 or PDAC-4662 cells were injected subcutaneously. When tumors were either palpable or seven days after tail vein injection, mice were randomized into either treadmill running or no exercise groups. B16F10 or PDAC-4662 tumor volumes were measured on the indicated days and are shown as the mean +/− S.E.M., *p < 0.05, *n* = 5–6 per group. (**B**) Tumor growth in exercised or non-exercised mice with or without chemotherapy. When B16F10 or PDAC-4662 tumors were palpable, wild type mice were treated with 2 mg/kg doxorubicin or 45 mg/kg gemcitabine weekly, with or without treadmill running. Tumor volume was measured on the indicated days and is shown as mean +/− S.E.M., *p < 0.05, *n* = 5 per group. (**C**) Tumor growth of PDAC-4662 flank tumors in mice treated with or without the anti-hypertensive drug and gemcitabine. PDAC-4662 tumor bearing mice were treated with prazosin, gemcitabine, or both. Tumor volumes were measured on the indicated days and are shown as mean +/− S.E.M, *n* = 3 for control, 4–5 for other groups, *p ≤ 0.01.

Exercise has been described as both pro- and anti-tumor growth in studies using mouse models [8, 15, 16]. Our data demonstrating vascular normalization in PDAC-4662 and B16F10 tumors should lead to increased blood delivery to the tumor promoting the growth of these tumors. Using moderate intensity treadmill exercise, we examined the growth of transplanted tumors in syngeneic wild-type mice with and without exercise. While exercise significantly promoted B16F10 melanoma growth, it had no significant effect on the growth of pancreatic ductal adenocarcinoma cells (PDAC-4662). Since aerobic exercise triggers cancer type specific effects on tumor growth, we examined how exercise affected tumorigenesis when combined with chemotherapy. The addition of aerobic exercise to chemotherapy inhibited both B16F10 and PDAC-4662 tumor growth significantly more than chemotherapy alone (Figure 2B and Supplementary Figure S1A, Supplementary Table S1). Exercise modulates multiple biological effects, including changes in metabolism, immune function, and vascular function. We detected no significant difference in tumor glucose, glutamate, glutamine, or lactate, and no difference in numbers of infiltrating CD45^+^CD11b^+^ or CD3^+^ cells (Supplementary Figure S1B–S1D). Therefore, to determine whether increasing blood flow is sufficient to enhance chemotherapeutic efficacy, we modulated blood flow pharmacologically. Anti-hypertensive drugs cause vasodilation by relaxing vascular smooth muscle cells to lower the pressure in large vessels. In normotensive mice, this leads to increased blood velocity in peripheral vessels such as tumor vessels, which lack vascular smooth muscle and do not dilate [17]. Combining the anti-hypertensive drug prazosin together with gemcitabine also increased gemcitabine efficacy towards PDAC-4662 tumors, suggesting that pharmacologically increasing blood velocity improves drug delivery to tumors (Figure 2C).

### Exercise improves delivery of chemotherapy to tumors

**Figure 3:**
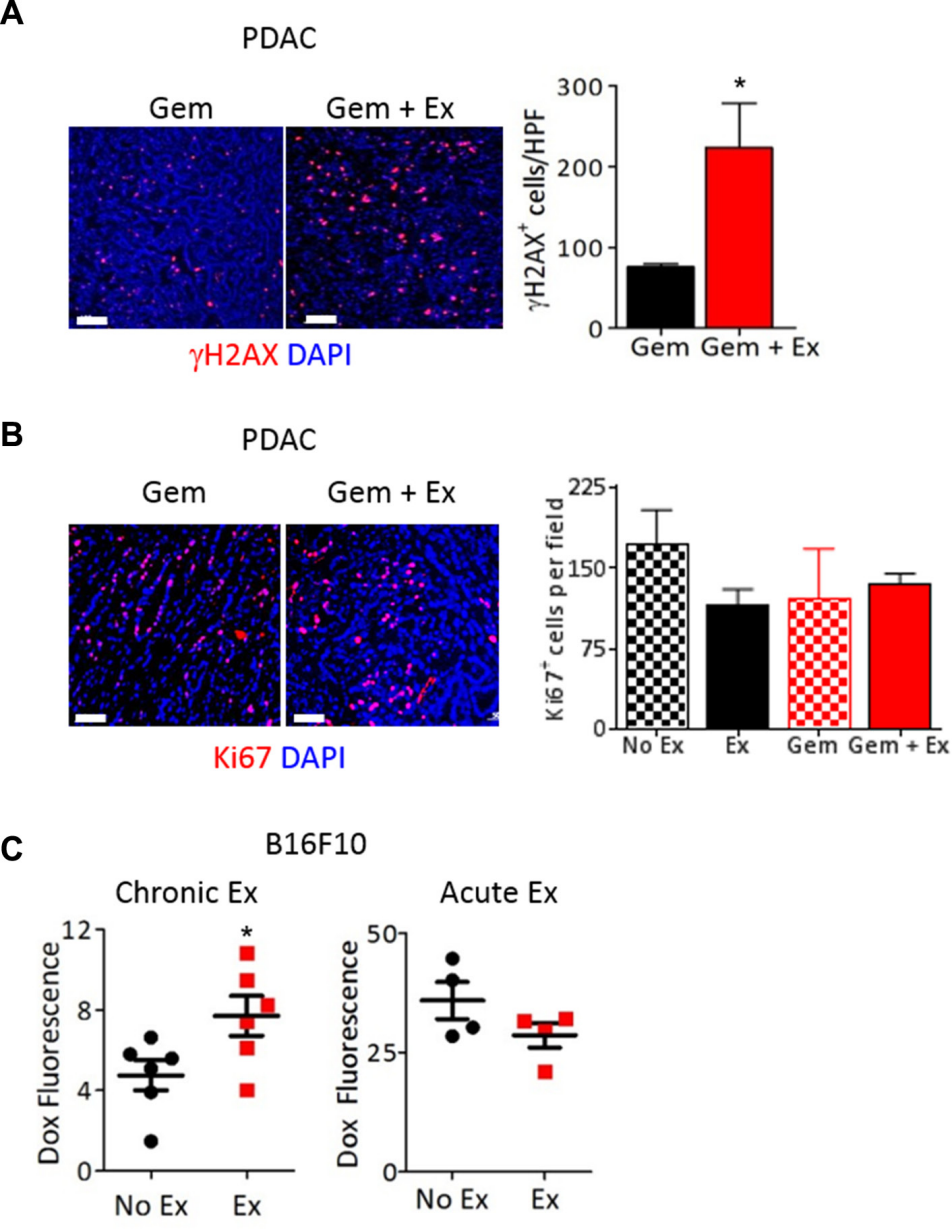
Exercise increases delivery of chemotherapy to the tumor (**A**, **B**) Representative images and quantification of (A) γH2AX (red) or (B) Ki67 (red) immunostaining on PDAC-4662 flank tumors harvested from mice after treatment with gemcitabine with and without exercise. Nuclei = blue, DAPI, *n* = 3–5 tumors per group, *p < 0.05, Bar = 50 μm (A) or 100 μm (B). (**C**) Quantification of doxorubicin in B16F10 flank tumors harvested from mice after chronic or acute exercise. B16F10 tumor-bearing mice were exercised for 2 weeks (chronic) followed by doxorubicin treatment 72 hours after exercise completion, then euthanasia. For acute exercise, mice were treated with doxorubicin when tumors were ∼100 mm^3^ then subjected to 1 session of treadmill running followed by euthanasia. Doxorubicin within tumors was quantified by spectrophotometry and normalized against tumors from mice that did not receive doxorubicin. *p < 0.05, *n* = 4 or 6 per group.

One explanation for the significant decrease in tumor growth observed upon combining chemotherapy with exercise in mice is greater drug delivery to tumors due to more normalized tumor vasculature. Consistent with this, we show increased expression of the DNA damage marker γH2AX in PDAC-4662 tumors from mice treated with exercise and gemcitabine compared to gemcitabine alone (Figure 3A) while there was no difference in tumor cell proliferation in tumors from mice treated with or without exercise or in tumor cells *in vitro* treated with serum from exercised or non-exercised mice (Figure 3B and Supplementary Figure S2A). Doxorubicin delivery was also significantly increased in B16F10 tumors from exercised mice even with a single dose of doxorubicin delivered after the final exercise session (Figure 3C). Our data suggests that chronic aerobic exercise normalizes tumor vasculature. To examine the necessity for vessel normalization versus simply increasing blood flow during acute exercise, B16F10 tumor-bearing mice were treated with doxorubicin immediately prior to undergoing a single exercise session. Unlike chronically exercised mice, there was no difference in doxorubicin levels in tumors from mice that underwent only one exercise session compared to non-exercised mice (Figure 3C).

### Increased blood flow alters the endothelial cell secretome to remodel tumor vasculature

**Figure 4:**
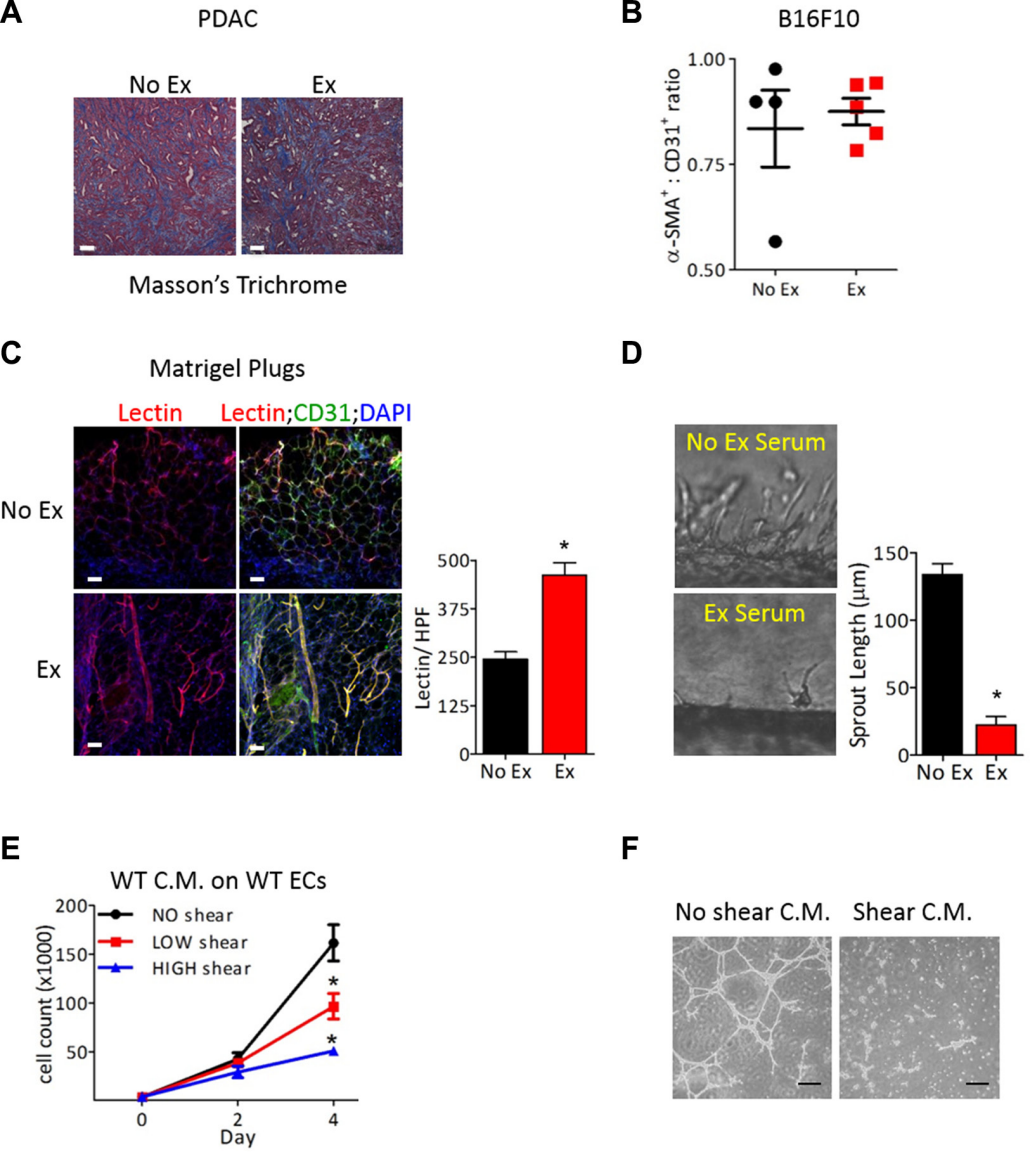
Exercise-induced tumor vascular remodeling is mediated in an endothelial cell autonomous manner (**A**) Representative image of Masson's trichome staining of PDAC-4662 tumors from exercised or non-exercised mice. (**B**) Quantification of for α-SMA and CD31 immunofluorescence of B16F10 tumors from exercised or non-exercised mice. Graph shows the average α-SMA:CD31 ratio +/− S.E.M for individual tumors, *n* = 4–5, bar = 100 μm. (**C**) Representative images and quantification of isolectin-B4 and CD31 in Matrigel plugs. Matrigel plugs with ECs harvested from mice after 7 days of exercise or non-exercise control were immunostained for isolectin-B4 (red), CD31 (green), and DAPI (blue), *n* = 4, * *p* < 0.05, Bars = 50 μm. (**D**) Representative images of EC sprouting in a microfluidic device *in vitro* after exposure to circulating media containing serum from exercised or non-exercised mice. Average sprout length was quantified after 24 hours, shown as the mean +/− S.E.M., *p < 0.05, *n* = 3. (**E**) Proliferation of naïve ECs at the indicated days in media conditioned by ECs after exposure to either no, low or high shear stress for 24 hours. Data are shown as mean +/− S.E.M., *p < 0.05, *n* = 3. (**F**) Images of Matrigel tube formation by naïve ECs in conditioned media harvested from ECs exposed to either no or high shear stress, Bar = 50 μm.

Mature blood vessels include both quiescent endothelium and pericyte coverage. However, the extent of desmoplastic stroma and pericyte coverage were similar in tumors from exercised and non-exercised mice, prompting us to focus on the endothelial response to exercise (Figure 4A, 4B). To examine the effects of exercise-increased blood flow specifically on ECs, we transplanted Matrigel plugs containing primary mouse lung ECs into mice. After a week of exercise, the vasculature in the Matrigel plugs from these mice was examined and showed elongated, large vessels with an almost 2-fold increase in lectin perfusion compared to Matrigel plugs from non-exercised mice (Figure 4C). Since previous studies have shown that tumor vascular normalization requires a restoration of the angiogenic balance in the tumor microenvironment [18], we examined whether the angiogenic secretome was altered due to increased blood flow by utilizing a microfluidic blood vessel model [19]. In this model, ECs form a lumenized tube through a collagen matrix and sprout outward into the collagen under controlled fluid flow, which mimics increased blood velocity that occurs during exercise (Supplementary Figure S2B). Addition of serum from tumor-bearing exercised mice, but not tumor-bearing non-exercised mice, significantly suppressed mouse lung EC sprouting and decreased the average sprout length by 83%, consistent with vascular maturation (Figure 4D). Similarly, serum from exercised but not non-exercised tumor-bearing mice inhibited EC growth and tube formation in Matrigel (Supplementary Figure S2C, S2D).

Serum contains angiogenic factors from several cellular sources besides ECs, and secretion of angiogenic factors occurs in response to numerous cues. To investigate the mechanical cues associated with exercise-increased blood flow, we exposed ECs to fluid flow at 0, 0.8, or 8 dynes/cm^2^ of shear stress in our microfluidic devices. We collected media after it passed through the EC lumen in these microfluidic devices. This shear stress-conditioned media was added to naïve ECs, and proliferation and tube formation were examined. While non- conditioned media had no effect on either proliferation or tube formation, the addition of media conditioned by ECs exposed to either low or high shear stress inhibited growth and tube formation of naïve ECs (Figure 4E, 4F).

### TSP-1 is upregulated after increased shear stress through activation of calcineurin-NFAT

**Figure 5:**
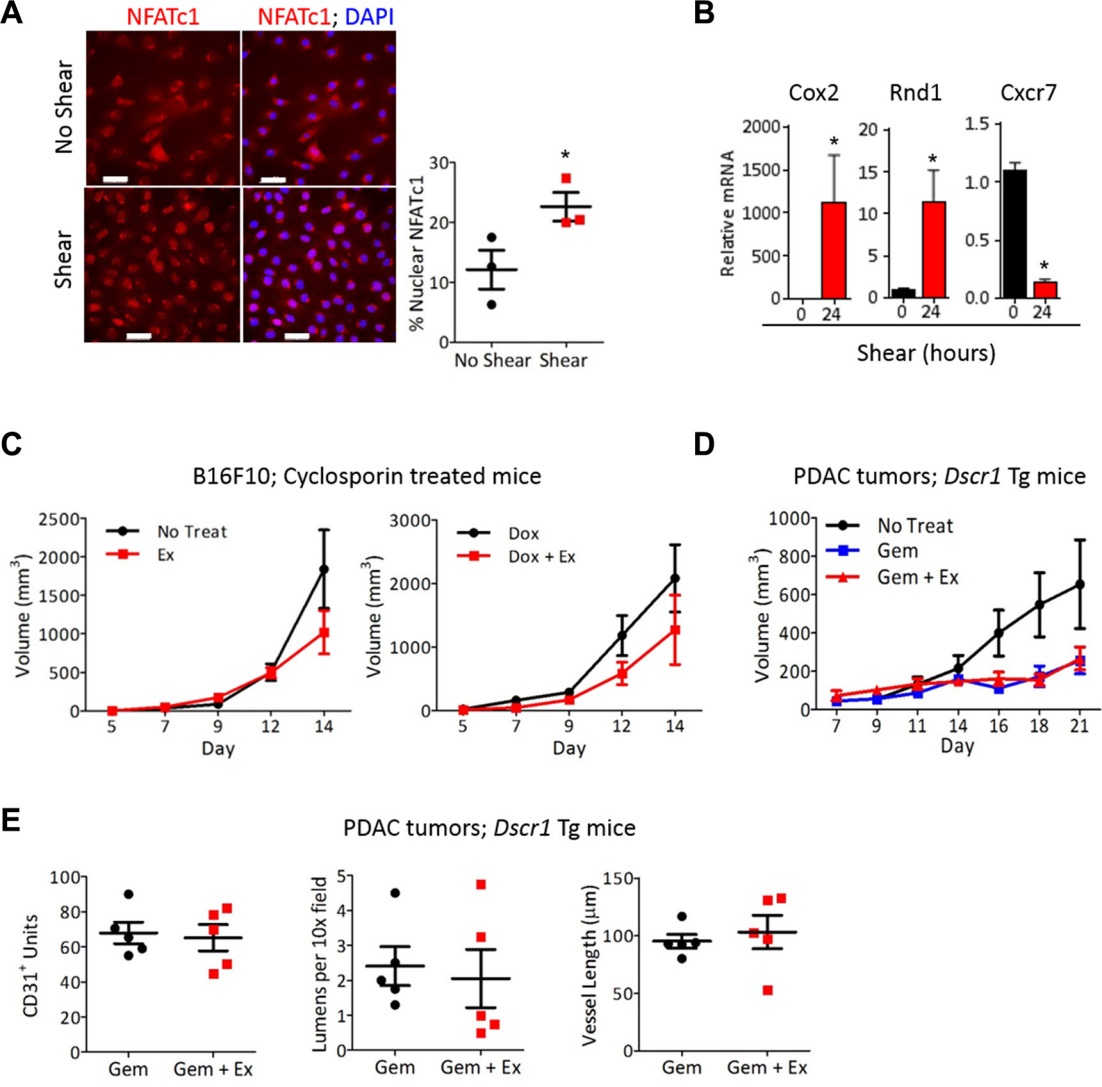
NFAT is necessary for vascular normalization by exercise-induced shear stress (**A**) Representative images of immunofluorescence for NFATc1 (red) and nuclei (DAPI;blue) on mLung ECs after exposure to shear stress for 8 minutes *in vitro*. The percent of nuclear NFATc1 was quantified for 4 random fields per sample to achieve one value per sample, and is shown as the mean +/− S.E.M *p < 0.05, *n* = 3, Bars = 50 μm. (**B**) qPCR analysis for *Cox2*, *Cxcr7*, and *Rnd1* in ECs exposed to 24 hours shear stress. mRNA normalized to 18s RNA, +/− S.E.M., *p < 0.05, *n* = 3. (**C**) B16F10 tumor volumes in wild type mice treated with cyclosporin A, with or without doxorubicin and treadmill running, as indicated. B16F10 cells were injected subcutaneously into WT mice and all mice were treated with cyclosporine A. After tumors were palpable, mice were randomized into either treadmill running or no exercise groups with or without 2mg/kg doxorubicin weekly. Tumor volumes were measured on the indicated days Data are mean +/− S.E.M., *n* = 5. (**D**) Tumor growth in *Dscr1* transgenic (Tg) mice. PDAC-4662 cells were injected subcutaneously into *Dscr1 Tg* mice. After tumors were palpable, mice were randomized into either treadmill running or no exercise groups. Tumor volumes were measured on the indicated days and are shown as the mean +/− S.E.M., *p < 0.05, *n* = 5 per group. (**E**) Quantification of vascular normalization. PDAC-4662 tumors from *Dscr1* Tg mice were assessed by immunofluorescence staining for microvessel density, lumens per field, and mean vessel length. 5 sections per tumor were quantified and used to obtain one value per tumor. Each marker represents one tumor, *n* = 5, not significant.

Screening both conditioned media from ECs after exposure to shear stress *in vitro* and serum harvested from tumor bearing-mice after aerobic exercise identified 4 angiogenic factors that were increased in both the conditioned media and serum (Supplementary Figure S2E). Three of these, VEGF-A, osteopontin, and MIP1a, are linked to the transcription factor Nuclear Factor of Activated T cells (NFAT). VEGF-A and osteopontin activate NFAT, while MIP1a is transcriptionally induced by NFAT [12, 20, 21]. We and others have previously shown that the calcineurin-NFAT pathway is a downstream mediator of VEGF signaling and plays a key role in EC activation [12, 22, 23]. Calcineurin, a ser/thr phosphatase activated by increased intracellular calcium, dephosphorylates the NFAT family of transcription factors leading to NFAT nuclear import [24]. Increased shear stress causes an influx of calcium and high intracellular calcium in ECs [25]. Exposure of ECs to shear stress increased calcineurin-NFAT activation as evidenced by NFATc1 nuclear localization (Figure 5A). Additionally, exposure of ECs to shear stress specifically increased the expression of the NFAT targets cyclooxygenase 2 and *Rnd1*, both of which have been previously reported to be shear responsive, but not the NFAT regulated gene *Cxcr7*, a gene unaffected by shear stress (Figure 5B). Inhibiting the calcineurin-NFAT pathway pharmacologically by treating B16F10 tumor bearing-mice with the specific calcineurin inhibitor cyclosporin A, prevented exercise-mediated increased tumor growth. Cyclsoporin A treatment also abrogated the increased efficacy of doxorubicin when combined with aerobic exercise (Figure 5C). Similarly, PDAC-4662 tumors grown in *Dscr1* transgenic mice [22], in which calcineurin-NFAT signaling is inhibited by increased expression of DSCR1, an endogenous inhibitor of calcineurin, showed no increased gemcitabine efficacy with the addition of exercise (Figure 5D) and no change in microvessel density, average vessel length, or total numbers of lumens (Figure 5E).

**Figure 6:**
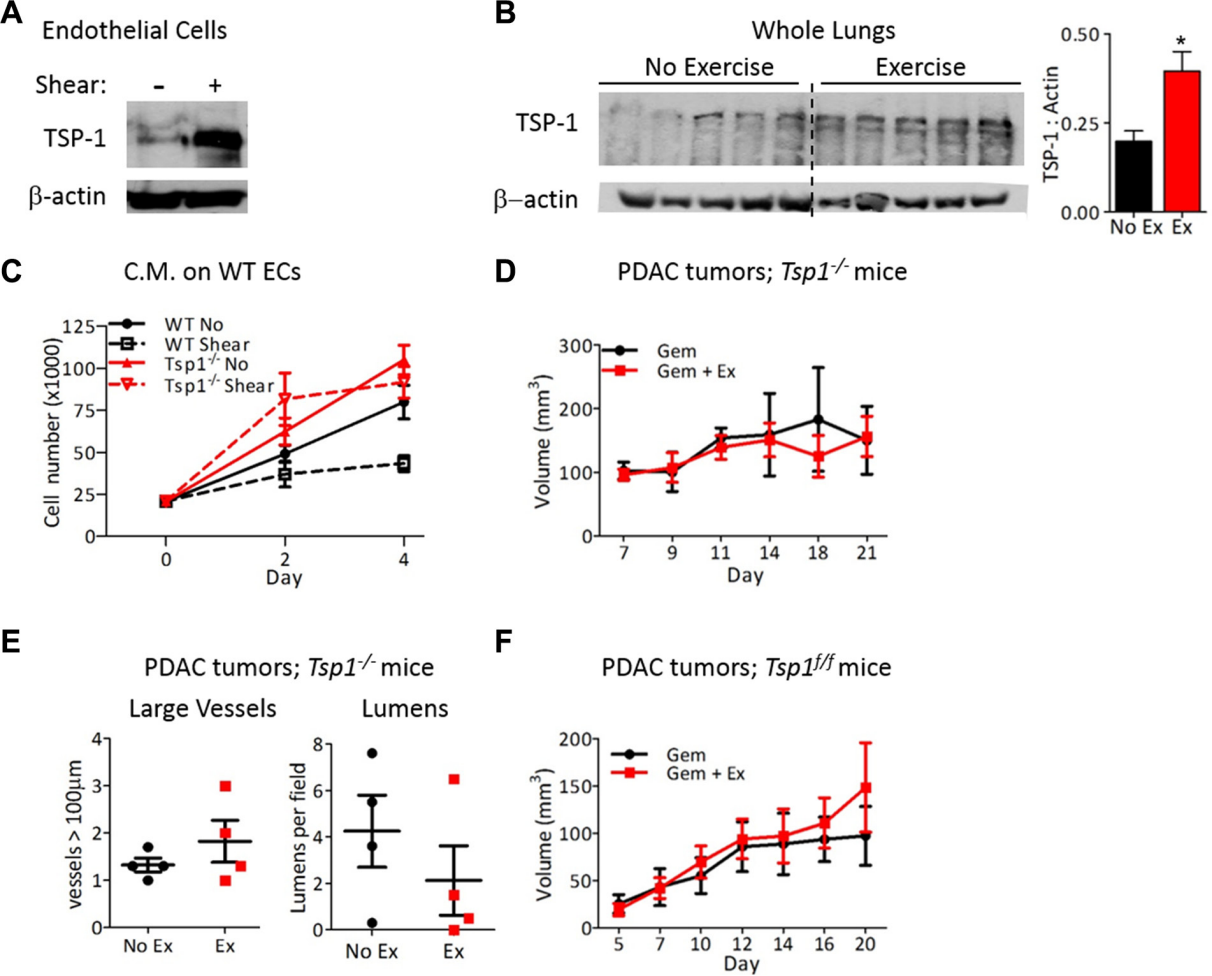
Endothelial-derived TSP-1 is critical for increased chemotherapeutic efficacy by shear stress (**A**) Immunoblots for TSP-1 expression by mouse lung ECs after exposure to 0 or 24 hours of shear stress (8 dynes/cm^2^). Actin was probed as a loading control. (**B**) Immunoblot for TSP-1 expression by lungs from wild-type mice exercised for two weeks with actin as a loading control. Each lane is a lung from one mouse. TSP-1 expression is quantified relative to actin, *n* = 5, *p < 0.05. (**C**) Proliferation of wild type or *Tsp1^−/−^* ECs in conditioned media from wild type or *Tsp1^−/−^* mLung ECs after exposure to 0 or 24 hours of shear stress. Data are represented as mean +/− S.E.M., *n* = 3, *p < 0.05. (**D**) Tumor growth in *Tsp1^−/−^* mice. PDAC-4662 cells were inoculated in the flanks of *Tsp1^−/−^* mice. Mice were treated with gemcitabine with or without exercise by the same protocol used in wild type mice for Figure 2. Tumor volumes shown as mean +/− S.E.M., *n* = 4, not significant. (**E**) Quantification of vascular normalization on tumors from *Tsp1^−/−^* mice. PDAC-4662 tumors from *Tsp1^−/−^* mice treated with exercise or non-exercise control were assessed by immunofluorescence staining for vessels >100 μm and the number of visible lumens per field. 5 sections per tumor were quantified and used to obtain one value per tumor. Each marker represents one tumor, *n* = 4, not significant. (**F**) PDAC-4662 flank tumor growth in *VE-cadherin-Cre; Tsp1^fl/fl^* mice. PDAC-4662 cells were inoculated into *VE-cadherin-Cre; Tsp1^fl/fl^* mice. When tumors were palpable, mice were treated with gemcitabine with or without exercise. Data are average tumor volumes +/−S.E.M. on the indicated days, *n* = 5, not significant.

Approaches to normalize tumor vasculature have primarily focused on decreasing pro-angiogenic proteins in the tumor microenvironment. However, upregulation of anti-angiogenic factors to restore the angiogenic balance has been shown to promote vascular normalization [26]. The potent anti-angiogenic protein thrombospondin-1 (TSP-1), is a direct transcriptional target of NFAT [27, 28] and was significantly upregulated in ECs after exposure to shear stress (Figure 6A) and in the lungs after chronic exercise (Figure 6B). Exposure of ECs to shear stress *in vitro* mimics the increased blood flow that occurs during exercise *in vivo*. In contrast to conditioned media from wild-type ECs, conditioned media from *Tsp1^−/−^* ECs after exposure to shear stress did not inhibit proliferation of naïve wild-type ECs (Figure 6C). The necessity for TSP-1 in exercise-induced tumor vessel normalization was evidenced by the lack of difference in chemotherapeutic efficacy with aerobic exercise in tumor bearing *Tsp1^−/−^* mice (Figure 6D; [29]) and the lack of vascular normalization in tumors from these mice (Figure 6E).

TSP-1 is expressed by numerous cell types, thus to specifically examine the contribution of EC-derived TSP-1 towards tumor vascular normalization, we generated *Tsp^fl/fl^* mice and bred them to VE-cadherin-Cre transgenic mice [30]. After confirmation of TSP-1 deletion specifically in ECs (Supplementary Figure S3A), we inoculated *Tsp^fl/fl^*; VE-cadherin-Cre mice with PDAC-4662 tumors and examined tumor growth during gemcitabine treatment with or without exercise. In these mice, the addition of exercise training during chemotherapy did not increase the efficacy of gemcitabine against PDAC tumors (Figure 6F), confirming the requirement for EC-derived TSP-1.

## DISCUSSION

Improved chemotherapy delivery through tumor vascular normalization has proven effective in multiple mouse models of cancer and in patients, but vascular normalization using anti-angiogenic agents has not gained wide clinical use due a small window of efficacy and significant adverse side effects [4, 16]. Therefore, normalizing tumor vasculature by modulating blood flow through aerobic exercise has clinical implications. While the importance of blood flow during developmental angiogenesis is appreciated, the role of shear stress created by flow during tumor angiogenesis is not well understood. Aerobic exercise has been shown to improve tumor vessel function and chemotherapy delivery in models of mammary and prostate cancer, but the underlying mechanisms mediating these effects are not known [8–10, 16]. We used moderate intensity treadmill running to demonstrate that exercise significantly enhances the efficacy of chemotherapy in mouse models of cancer due to vascular normalization. Increased blood flow due to aerobic exercise triggered the remodeling of tumor vessels to a more functional state. We identify a previously unknown shear stress-responsive pathway in ECs, calcineurin-NFAT-TSP-1 signaling, and demonstrate that TSP-1 expression is critical for exercise-induced tumor vascular normalization.

Aerobic exercise alone increased the rate of B16F10 melanoma growth yet had modest effects on the growth of PDAC tumors. This is consistent with reports of tumor-specific effects of exercise on tumor growth, where exercise alone was sufficient to inhibit mammary carcinoma growth but had no effect on prostate tumor growth in mice [10, 16]. However, importantly, exercise in combination with chemotherapy showed enhanced anti-tumor efficacy of both tumor types. Using aerobic exercise as a non-pharmacologic method for remodeling tumor vasculature may circumvent the challenges associated with anti-angiogenic therapies currently in clinical use. In addition to the associated adverse side effects [31], pharmacologic angiogenesis inhibitors have a small therapeutic window; low doses are ineffective and high doses deplete too many vessels leaving insufficient vasculature. Over-pruning of vessels impedes drug delivery and increases tumor hypoxia, often making tumor cells more aggressive and possibly increasing metastasis [32]. In contrast, it is unlikely that exercise will over-prune tumor vasculature because blood vessels respond to chronic exercise by maturing and then becoming quiescent [33].

Prior studies have demonstrated that exercise increases blood perfusion to tumors [9, 10], however the mechanism by which vascular remodeling occurs is not understood. Our work indicates that normalization of tumor vasculature underlies the increased tumor blood flow during aerobic exercise. Using an *in vitro* model of shear stress to mimic increased blood flow by exercise, we identified activation of the transcription factor NFATc1 in ECs as important for vascular remodeling in response to shear stress. We found that in response to increased shear, activation of calcineurin-NFAT signaling in ECs upregulates the anti-angiogenic protein TSP-1. Altering the expression of secreted angiogenic factors allows cross-talk between ECs experiencing shear stress and ECs in regions of disrupted shear, promoting vascular normalization. Indeed, conditioned media from ECs exposed to shear stress or serum from exercised mice inhibited proliferation of naïve ECs in a TSP-1 dependent manner.

To date, most studies of vascular normalization have focused on decreasing pro-angiogenic factors produced by many cell types in the tumor microenvironment [34]. However, it was unknown whether modulation of angiogenic factors produced by ECs alone would be sufficient to overcome the pro-angiogenic growth factor milieu secreted by tumor cells. Further, increasing endogenous anti-angiogenic protein expression to restore the angiogenic balance has not been explored. We generated transgenic mice with EC specific loss of *Tsp1* to demonstrate that EC-derived TSP-1 is necessary for tumor vascular normalization in response to exercise.

Exercise did not improve chemotherapeutic efficacy in mice lacking *Tsp1* systemically or specifically in the endothelium. Tumors grown in these mice had more open lumens than tumors from wild type mice even without treatment, consistent with previous reports of dilated tumor vessels in *Tsp1^−/−^* mice [35]. This suggests the possibility that vessels in *Tsp1^−/−^* mice do not respond to exercise because they are already maximally dilated, although vessel structure does not always correlate with function in tumors. Endothelial TSP-1 may be necessary to mediate exercise-induced changes in vessel function independent of its effect on vessel dilation or lumens.

Moderate intensity treadmill running in mice, roughly the equivalent of brisk walking in humans at approximately 60–70% V0_2_ max [36], enhanced the efficacy of chemotherapy. The addition of exercise during chemotherapy allowed a 50% reduction in the dose of gemcitabine while retaining the same anti-tumor efficacy as standard gemcitabine dosing. Together with other studies demonstrating safety and feasibility of exercise regimens for patients undergoing cancer treatment, our findings represent a mechanistic explanation of an easily translatable intervention that is likely to have large clinical impact.

## MATERIALS AND METHODS

### Cell culture

B16F10 cells were from ATCC. PDAC-4662 were isolated from pancreatic tumors of *Kras*^LSL-G12D/^*;Trp53*^LSL-R172H/+^;*Pdx1-Cre* (KPC) mice and were provided by Dr. Robert Vonderheide (University of Pennsylvania School of Medicine). Primary mouse lung endothelial cells were isolated from mice using CD31 magnetic bead positive selection, as described previously [28]. Culture conditions described in supplemental methods. Exposure to fluid flow using the microfluidics lumenized endothelial cell device has been described [19]. Cells were exposed to 0.8 (low) or 8.0 (high) dynes/cm^2^ of shear stress for 24 hours. Conditioned media was collected after flowing through the lumen once. For RNA or protein harvest and for immunofluorescence, cells were exposed to fluid flow (∼10 dynes/cm^2^ of shear stress) for the indicated time using a modified 100 mm plate in which cells are plated only on the outer rim and placed on an orbital rotator plane, as described in [37].

### Animal experiments

All animal experiments were approved by the University of Pennsylvania Institutional Animal Care and Use Committee. Mice were 6–8 weeks old and sex-matched within each experiment. Wild-type mice were bred from C57Bl/6J obtained from Jackson Laboratories. *Dscr1* transgenic mice on a C57Bl/6 background are described in [22]. *Tsp1^−/−^* mice on a C57Bl/6 background were obtained from Dr. Jack Lawler (Beth Israel Deaconess Medical Center, Boston, MA) [29]. *VE-Cadherin*–Cre mice were obtained from Dr. Luisa Iruela- Arispe [30].

300,000 tumor cells in 200 μl PBS were injected subcutaneously into the flanks of mice. When tumors reached ∼35 mm^3^ (4–7 days post injection) or at indicated volume, mice began treatment. For PDAC tumor-bearing mice, the exercise and exercise plus chemotherapy groups performed 45 minutes of treadmill running for 5 consecutive days per week at 12 m/min. For PDAC tumor-bearing mice, gemcitabine was delivered by tail vein injection 3 times per week. For B16F10 tumor-bearing mice, the exercise and exercise plus doxorubicin groups performed 45 minutes of treadmill running for 5 consecutive days per week at 10 m/min. Mice received 2 mg/kg of doxorubicin by tail vein injection once per week. At the end of each experiment, 72 hours after the final exercise session, mice were euthanized, tumors harvested and fixed in formalin or frozen in OCT. Mice were given prazosin hydrochloride (50 mg/L in water) (Sigma) beginning on the day of tumor inoculation with prazosin replaced every other day. Cyclosporin A (10 mg/kg in peanut oil) (Teva Czech Industries) was delivered daily by oral gavage beginning 5 days before tumor cell implantation. Statistical analysis for all experiments is explained in Supplemental Methods.

### Doxorubicin quantification

Mice were inoculated with B16F10 tumors and injected with 10 mg/kg doxorubicin via tail vein 72 hours after the last exercise session. Twenty minutes later, mice were euthanized and tumors were immediately harvested, weighed, and homogenized in acid isoproponal/Triton-X as described [38]. Doxorubicin fluorescence was read using a spectrophotometer (λ = 488) and compared to a standard curve, then normalized against the weight of tumor input and the background reading of a tumor from a mouse that received no doxorubicin.

### Immunofluorescence staining

Detailed methods can be found in supplemental methods. Antibodies include: rat anti-mouse CD31, 1:50 (BD Pharmingen); mouse anti-mouse alpha smooth muscle actin, 1:100, (Abcam); rabbit anti-mouse γH2AX, 1:750 (Millipore); rabbit anti-mouse Ki67, 1:250 (Novus); rabbit anti-human CD3, 1:200 (Dako).

For quantification, 5 random 10× magnification pictures were taken of each slide and the area of CD31^+^ structures, number of visible lumens, vessels, and vessels > 100 μm was counted. Values for each of the 5 sections were averaged to obtain one value for each tumor. Individual averages for all tumors within a treatment group were then averaged to determine the group average and SEM.

### Matrigel tube formation assay

WT and *Tsp1^−/−^* mouse lung ECs were exposed to shear stress using our lumenized EC tube device (described above) for 24 hours. Conditioned media was collected, centrifuged, and used for EC tube formation assays. Detailed methods for tube formation assay and Matrigel in mice can be found in supplemental methods.

## SUPPLEMENTARY MATERIALS FIGURES AND TABLE




