# Supplementary material for: Tumor vessel normalization after aerobic exercise enhances chemotherapeutic efficacy
Source: Oncotarget. 2016 Aug 31;7(40):65429–40. doi: 10.18632/oncotarget.11748 (PMC5323166; doi:10.18632/oncotarget.11748)
Supplement: Supplementary file 1 [file oncotarget-07-65429-s001.pdf]

Tumor vessel normalization after aerobic exercise enhances chemotherapeutic efficacy

Supplementary Materials

SUPPLEMENTARY METHODS

Cell culture

B16F10 and PDAC-4662 cells were cultured in DMEM (Invitrogen) containing 10% Fetal Bovine Serum, 2 mM glutamine, 100 U/mL penicillin, and 100 µg/mL streptomycin. B16F10 cells were authenticated by short tandem repeat fingerprinting, IDEXX RADIL at University of Missouri, Columbia, MO; 2011. ECs were cultured in Advanced DMEM (Invitrogen) containing 15% Fetal Bovine Serum, 50 mg heparin, 12.5 mL of 1 M HEPES, 2 mM glutamine, 100 U/mL penicillin, 100 µg/mL streptomycin, and one bottle of endothelial cell mitogen (Biomedical Technologies), and were used between passage 3 and 7.

Western blotting

For Western blotting, membranes were incubated with TSP-1 antibody clones SPM-321 and A6.1 (1:500; Santa Cruz Biotechnology) or β-actin antibody (1:3,000; Sigma) followed by horseradish peroxidase–labeled secondary antibodies, and detected using enhanced chemiluminescence solution. Densitometry analysis was performed on minimally exposed blots using ImageJ software (http://rsbweb.nih.gov/ij/) to determine a TSP-1:actin ratio.

Immunofluorescence staining

Paraffin embedded slides were de-paraffinized followed by antigen retrieval in 200 mg/mL proteinase K at 37°C for 40 minutes. Frozen sections were fixed in ice cold acetone for 10 minutes. Slides were washed 3 times in PBS, and incubated with primary antibodies diluted in 10% normal goat serum overnight at 4°C. AlexaFluor594 or AlexaFluor488 secondary antibodies were used at 1:1500 for 1 hour at RT. Nuclei were stained wtih Hoechst 33342 (Life Technologies). Images were taken with a digital camera AxioCAM HRc (Zeiss) mounted on Zeiss Imager M1 Axio using Zeiss AxioVision Acquisition software (version 4.5).

For NFAT staining, cells were fixed for 15 minutes in 4% paraformaldehyde, permeabilized using 0.4% triton-X then incubated with anti-mouse NFATc1 (Santa Cruz Biotechnology, 1:50) for 3 hours at RT, then washed and incubated with goat ant-mouse Alexa594 (Life Technologies, 1:1500) for 30 minutes at RT.

Matrigel tube formation assays and matrigel in mice

12 well culture dishes were coated with a 1:1 mixture of ice cold RPMI:matrigel (BD Biosciences). Matrigel was hardened at 37°C for 45 minutes. ECs were resuspended in the appropriate conditioned media at a final concentration of 1.25×10^5 cells per 2 mL media then added on top of the matrigel and tube formation was documented over time. Assays were performed in 3 independent experiments.

For in vivo matrigel experiments, 500,000 wild type mouse lung ECs were mixed into 500 µl of ice cold matrigel. Matrigel was implanted subcutaneously in wild type mice. The following day mice were either not exercised or began daily treadmill running for 45 minutes/day at 12 meters/min. After 7 days mice were injected with isolectin-B4 (Invitrogen) by tail vein and euthanized after 20 minutes. Matrigel plugs were harvested, fixed in 4% paraformaldehyde, sectioned then immunostained.

Statistical analysis

Statistical Analysis was performed using GraphPad Prism 6 software. For tumor growth experiments comparing only two treatment groups or for vessel analysis comparing two groups, Mann-Whitney test or Student's *T* test was used. For analysis of tumor growth experiments comparing multiple groups (i.e. combination chemotherapy and exercise), Two-Way ANOVA and Tukey's Multiple Comparisons Tests were used.

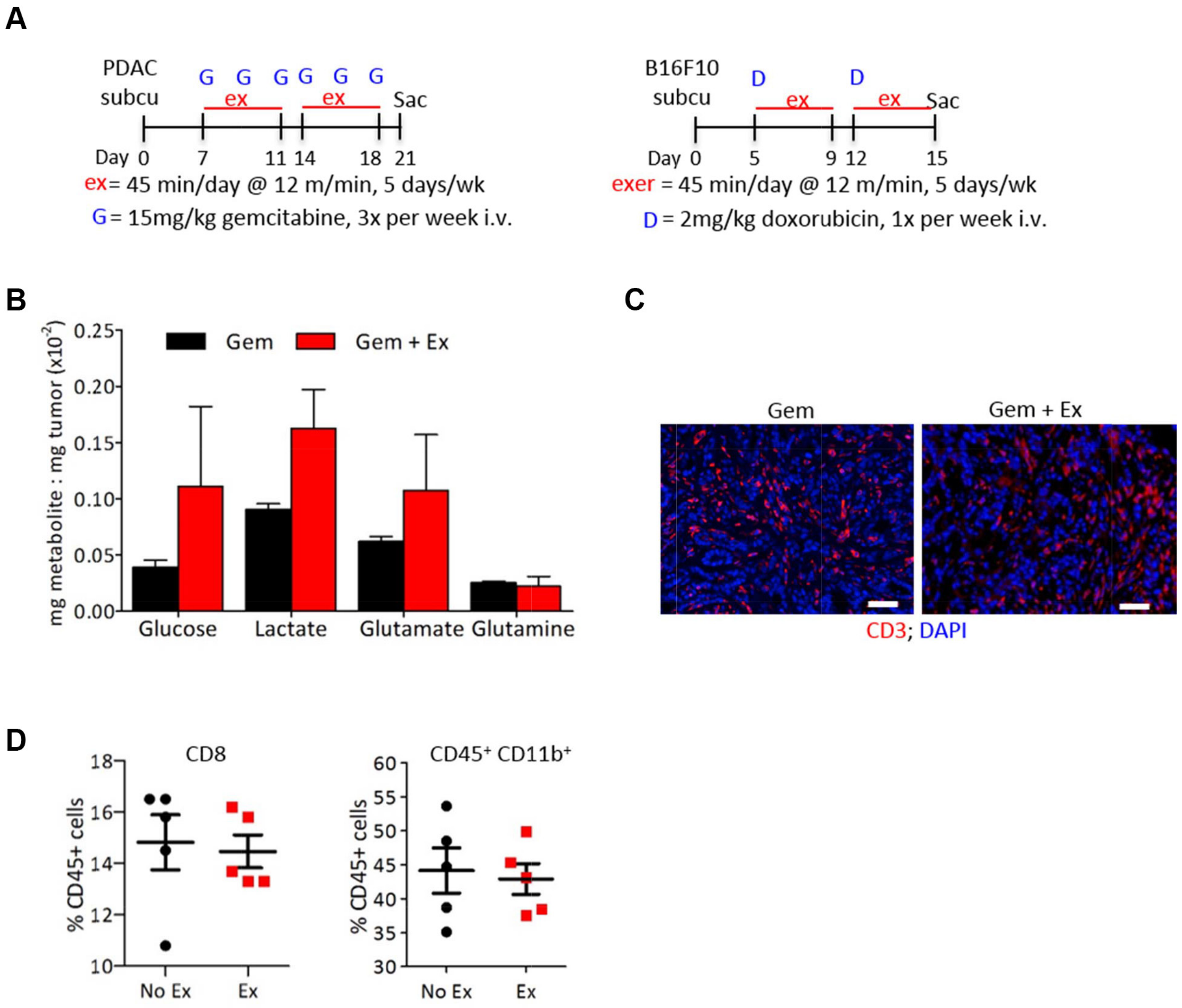

Supplementary Figure S1: Exercise serum does not change tumor cell proliferation *in vitro*. (**A**) Experimental design for exercise and chemotherapy combination experiments. (**B**) YSI metabolite analysis of PDAC-4662 homogenates from gemcitabine or gemcitabine plus exercise treated mice, *n* =3. (**C**, **D**) Wild type mice were inoculated with PDAC-4662 cells. When tumors were palpable, mice were treated with or without daily treadmill run ning. After two weeks, tumors were collected and halved. (C) Tumors were analyzed by immunofluorescent staining for CD3 (red) and nuclei (blue), or (D) tumor homogenates were analyzed by flow cytometry for $CD45^{+}CD8^{+}$ and $CD45^{+}CD11b^{+}$ cells. Each marker represents one tumor, *n* = 5.

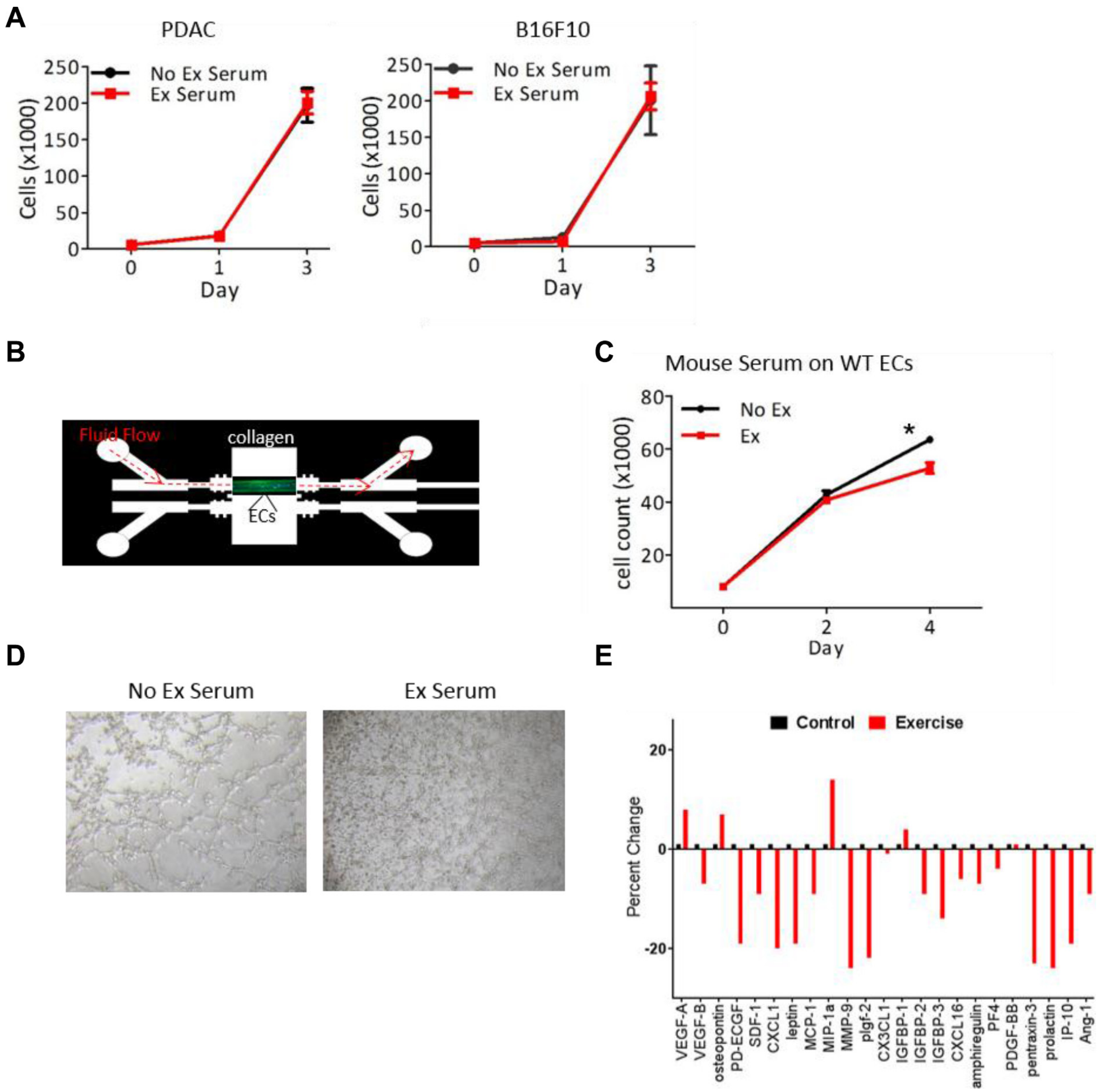

Supplementary Figure S2: Exercise changes the angiogenic secretome. (**A**) Cell proliferation is not changed by the addition of serum from exercised or non-exercised mice. PDAC-4662 or B16F10 cells were plated in media containing 5% serum from either exercised or non-exercised mice. Mean cell number per well +/– SEM over time are shown. $N = 3$ per group. (**B**) Diagram of the microfluidic device used to induce shear stress. ECs are plated in a collagen bed to form an open lumen through which media is passed. (**C**) Wild type EC proliferation over time in mouse. Wild type mLung ECs were plated in 5% serum from exercised or non-exercised mice. Mean cell number over time is shown, $*p < 0.05$, $n = 3$ per group. (**D**) Images of Matrigel tube formation by naïve ECs in serum collected from mice from either control or exercise groups, Bar = 50 µm. (**E**) Differences in secreted angiogenic factors were compared using an antibody array. Serum from tumor bearing exercised or non-exercised control mice was analyzed using an angiogenic antibody array. Graph shows factors that were different between non-exercise and exercise serum, $n = 2$ per group.

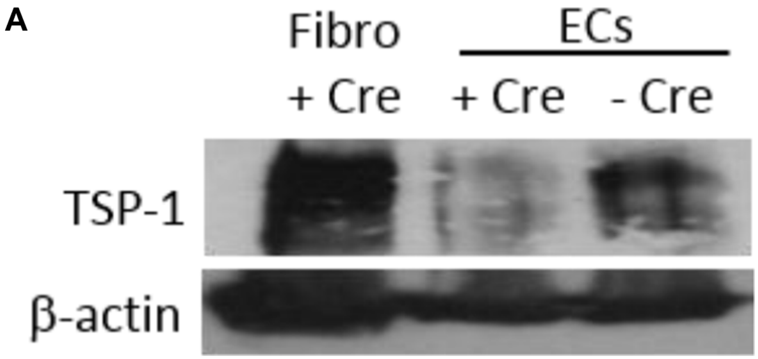

Supplementary Figure S3: Tsp1 is deleted in ECs from *Tsp1$^{f/f}$*; *VE-cadherin*-Cre mice. (**A**) Immunoblot for TSP-1 and actin. Lung fibroblasts or ECs were isolated from *Tsp1$^{f/f}$*; *VE-cadherin*-Cre or *Tsp1$^{f/f}$* mice and immunoblotted for TSP-1 and actin. mLung EC population was ~80% ECs.

Supplementary Table S1: Combination of exercise and chemotherapy increases the efficacy of chemotherapy in melanoma and pancreatic tumor models. See Supplementary_Table_S1
