# Supplementary material for: Tumor vessel normalization after aerobic exercise enhances chemotherapeutic efficacy
Source: Oncotarget. 2016 Aug 31;7(40):65429–40. doi: 10.18632/oncotarget.11748 (PMC5323166; doi:10.18632/oncotarget.11748)
Supplement: Supplementary file 2 [file oncotarget-07-65429-s002.docx]

**PDAC-4662 Tumor Growth Statistics; complementary data to Figure 2b**

| **Tukey's multiple comparisons test** | | | | | | | | | |  |
| --- | --- | --- | --- | --- | --- | --- | --- | --- | --- | --- |
|  | | **Mean Diff.** | **95% CI of diff.** | | **Significant?** | | **Summary** | | **P value** | |
| DAY 18 | |  |  | |  | |  | |  | |
| Gem + Ex vs. Gem | | -227.9 | -409.5 to -46.26 | | Yes | | ** | | <.01 | |
| Gem + Ex vs. Ex | | -351.3 | -525.2 to -177.4 | | Yes | | **** | | < 0.0001 | |
| Gem + Ex vs. No Ex | | -284 | -465.7 to -102.4 | | Yes | | *** | | <.001 | |
| Gem vs. Ex | | -123.4 | -297.3 to 50.50 | | No | | ns | |  | |
| Gem vs. No Ex | | -56.14 | -237.8 to 125.5 | | No | | ns | |  | |
| Ex vs. No Ex | | 67.27 | -106.6 to 241.2 | | No | | ns | |  | |
| DAY 21 | |  |  | |  | |  | |  | |
| Gem + Ex vs. Gem | | -256.7 | -438.4 to -75.09 | | Yes | | ** | | <.01 | |
| Gem + Ex vs. Ex | | -541.4 | -715.3 to -367.5 | | Yes | | **** | | < 0.0001 | |
| Gem + Ex vs. No Ex | | -679.2 | -860.9 to -497.6 | | Yes | | **** | | < 0.0001 | |
| Gem vs. Ex | | -284.7 | -458.6 to -110.8 | | Yes | | *** | | <.001 | |
| Gem vs. No Ex | | -422.5 | -604.1 to -240.9 | | Yes | | **** | | < 0.0001 | |
| Ex vs. No Ex | | -137.8 | -311.8 to 36.06 | | No | | ns | |  | |
| **Two-way ANOVA** | | | | | | | | | |  |
| **Source of Variation** | **% of total variation** | | | **P value** | | **P value summary** | | **Significant?** | |  |
| Interaction | 14.2 | | | < 0.0001 | | **** | | Yes | |  |
| Difference over time | 53.64 | | | < 0.0001 | | **** | | Yes | |  |
| Difference between groups | 11.03 | | | < 0.0001 | | **** | | Yes | |  |

**B16F10 Tumor growth statistics; complementary data to Figure 2b**

| **Tukey's multiple comparisons test** | | | | | |
| --- | --- | --- | --- | --- | --- |
|  | Mean Diff. | 95% CI of diff. | Significant? | Summary | P value |
| DAY 15 |  |  |  |  |  |
| No Ex vs. Ex | -561.2 | -888.8 to -233.5 | Yes | *** | <.01 |
| No Ex vs. Dox | 148.8 | -178.8 to 476.4 | No | ns |  |
| No Ex vs. Dox + Ex | 532.2 | 182.9 to 881.4 | Yes | *** | <.001 |
| Ex vs. Dox | 710 | 367.8 to 1052 | Yes | **** | < 0.0001 |
| Ex vs. Dox + Ex | 1093 | 730.4 to 1456 | Yes | **** | < 0.0001 |
| Dox vs. Dox + Ex | 383.4 | 20.40 to 746.3 | Yes | * | <0.05 |

| **Two-way ANOVA** | | | | |
| --- | --- | --- | --- | --- |
| **Source of Variation** | **% of total variation** | **P value** | **P value summary** | **Significant?** |
| Interaction | 11.54 | < 0.0001 | **** | Yes |
| Difference over time | 54.65 | < 0.0001 | **** | Yes |
| Difference between groups | 8.944 | < 0.0001 | **** | Yes |
